# Recovery of fuel from real waste oily sludge via a new eco-friendly surfactant material used in a digital baffle batch extraction unit

**DOI:** 10.1038/s41598-023-37188-9

**Published:** 2023-06-19

**Authors:** Jasim I. Humadi, Saad A. Jafar, Nisreen S. Ali, Mustafa A. Ahmed, Mohammed J. Mzeed, Raheem J. Al-Salhi, Noori M. Cata Saady, Hasan Sh. Majdi, Sohrab Zendehboudi, Talib M. Albayati

**Affiliations:** 1 Department of Petroleum and Gas Refining Engineering, College of Petroleum Processes Engineering, Tikrit University, Tikrit, Iraq; 2 Materials Engineering Department, College of Engineering, Mustansiriyah University, Baghdad, Iraq; 3 Ministry of Oil, North Refineries Company, Baiji Refinery, Slah Al-Deen, Iraq; 4 Department of Civil Engineering, Memorial University of Newfoundland, St. John’s, NL A1B 3X5 Canada; 5 Department of Chemical Engineering and Petroleum Industries, Al-Mustaqbal University, Babylon, 51001 Iraq; 6 Department of Chemical Engineering, University of Technology-Iraq, 52 Alsinaa St., P.O. Box 35010, Baghdad, Iraq

**Keywords:** Environmental chemistry, Engineering

## Abstract

This study focused on developing a new cocktail extraction agent (CEA) composed of solvent and a new surfactant material (SM) for enhancing the efficiency of fuel recovery from real waste oil sludge (WSO). The effects of different solvents (e.g. methyl ethyl ketone (MEK), naphtha, petrol and kerosene), SMs (Dowfax and sodium thiosulfate), extraction time (10–20 min), extraction temperatures (20–60 °C) and CEA/sludge ratios (1–4) on the extraction performance were investigated. SMs and DBBE design enhanced the extraction efficiency by increasing the dispersion of solvent in WSO and enhancing the mixing and mass transfer rates. Results proved that Dowfax was the best SM for oil recovery under various conditions. The best CEA (e.g. MEK and Dowfax) provides the maximum fuel recovery rate of 97% at a period of 20 min, temperature of 60 °C and 4:1 CEA/sludge ratio. The produced fuel was analysed and fed to the distillation process to produce diesel oil. The characteristics of diesel oil were measured, and findings showed that it needs treatment processes prior its use as a finished fuel.

## Introduction

Fossil fuel is continuously depleted because of the growing demand in different sectors, such as in agriculture and transportation, and the escalation of oil prices in the international market. Therefore, the search for alternative fuel sources has become urgent^1–5^. Oily sludge is produced in different petroleum processes, such as transportation, storage and refining processes. The Iraqi Baiji Refineries produce an annual average of 3000–3500 m^3^/year of waste oil sludge (WSO), which is sent to landfills for natural attenuation without treatment technology. The annual global productivity of WSO is above 60 million tons^6–8^. It occupies volume and reduces efficiency when it settles in petroleum storage tanks and transportation tankers. The high viscosity and solid material contamination of oily sludge, such as sand and rust, lead to severe and harmful problems if introduced into refinery streams^9–13^. Oily sludge includes hydrocarbon compounds, water, solid content, heavy materials and water in oil emulsion. Additionally, it has a dangerous influence on the environment and human health because it includes toxic and carcinogenic materials^14–16^. Conventional technologies of sludge oil treatment include incineration, landfilling, solidification physical (solvent extraction), chemical (pyrolysis), and combined chemical and physical methods^7,17,18^. Common landfilling method has disadvantages, such as high cost, thereby making disposal problematic and unacceptable due to increases in stringent environmental regulations. Therefore, interest in finding alternative technologies for eco-friendly oily sludge treatments is significant^7,19^. The solvent extraction process (SEP) has effective benefits in treating WSO more thoroughly and has the ability to recover most of hydrocarbon compounds^20–22^. Based on economic and environmental reasons, the recovery of valuable fuel from oily sludge via SEP is attractive and has been tested and operated commercially^23^. Thus, this is the key to searching for new extraction agent cocktails based on the nature of oily sludge, such as solvents combined with surfactant materials (SMs) with high performance and low cost. The extraction unit design can differ with the type of process. The geometric design of an extraction unit in the absence of baffles result in fuel transfer along circular paths with high severe circumferential velocity, hence causing poor and undesirable mixing and creating a vortex at the free surface. Installing the baffles in the unit successfully devastates the rotating flows of fuel, thereby prohibiting the creation of vortex so the fuel surface will be almost flat. Moreover, axial flow patterns become highly severe, which promotes the mixing rate^24,25^. Taiwo and Otolorin (2016) investigated the performance of single and cocktail solvents during the extraction of the tank bottom sludge. The results showed that 66.25% of oil was recovered^26^. Nezhdbahadori et al. (2018) studied the activity of MEK and toluene as polar and nonpolar solvents in the extraction of fuel from WSO. The results proved that 30.41% and 37.24% of oil were recovered for MEK and toluene, respectively^14^. Al-Doury (2019) studied the extraction performance for oily sludge treatment using light naphtha, heavy naphtha, kerosene, petrol and MEK as solvents. The results proved that MEK achieved 95% fuel extraction efficiency at an extraction temperature of 60 °C^16^.

The traditional extraction process for the sludge oil used only solvent to recover the fuel in traditional extraction unit. In this study, the new composite of the solvent and new surfactant material and the modified design of the extraction unit remarkably enhanced the efficiency of fuel recovery from the real waste sludge oil. The oily sludge was collected from the oil storage tanks of Baiji Refineries in Iraq. Digital baffle batch extraction (DBBE) unit designed for recovering fuel from WSO. New cocktail extraction agents (CEAs) (e.g. solvent/Dowfax and solvent/thiosulfate [STH]) are used to achieve the best performance in recovering fuel from the oily sludge produced from the Iraqi Baiji Refineries. The solvent extractive process (SEP) is achieved to determine the performance of new CEAs in the modified design of the digital baffle batch extraction (DBBE) unit under moderate experimental parameters with different variables (e.g. extraction temperature, extraction time, sludge-to-oil ratio and CEA type).

## Experimental work

### Materials

**Figure 1:**
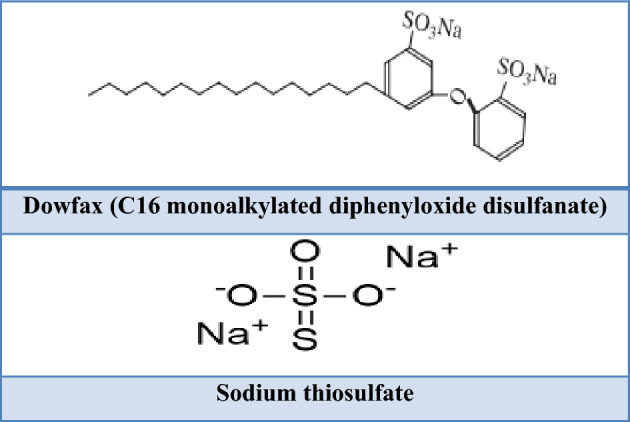
The chemical structures of surfactant materials.

The WSO employed in the present study was collected from the bottom of crude oil storage tanks in the Iraqi North Oil Company (NRC.)/Baiji Refineries/Salah al-Din. Subsequently, WSO was kept under room conditions for the duration of this work without pre-treating process. Methyl ethyl ketone (MEK), naphtha, petrol and kerosene were used as solvents in this work. The three types of oil solvents were provided by the Iraqi North Oil Company (NRC.)/Baiji Refineries/Salah al-Din. The physical properties of these fuels are explained in Table 1**.** MEK was provided by the THOMAS BAKER Company in India. The characteristics of MEK are explained in Table 2. STH and Dowfax (C_16_ monoalkylated diphenyloxide disulfanate) were used as SMs without further treatment. STH (AnalaR, purity of 99.5%) was provided by the THOMAS BAKER Company in India. Dowfax (35% active) was provided by the Dow Chemical under the trade name ‘Dowfax 8390’. The chemical structures of the SMs are illustrated in Fig. 1.

**Table 1:** Physical characteristics of oil solvents.

Physical property	Naphtha	Gasoline	Kerosene
Density @15 °C	703.4	731.1	790.9
API at 60F	69.7	60	47.4
R. V. P. (psi)	6.7	8.1	–
Total sulfur, (ppm)	665	9.3	14
Distillation, (°C)			
Final boiling point, IBP (°C)	40	41	168
5%	50	47	177
50%	85	98	203
90%	135	146	228
Final boiling point, FBP (°C)	169	187	248

**Table 2:** Specifications of methyl ethyl ketone.

Properties	MEK
Density (g/cm^3^)	0.81
Chemical formula	CH3COCH2CH3
Molecular weight (g/mol)	72.11 g/mol
Physical state	Liquid
Purity	100%

### Preparation of CEAs

SM was added at the desired concentration into the solvent to prepare CEAs. During the preparation of each CEA type, 0.25 gm of SM was added to 150 gm of solvent. The extracting cocktail was stirred via a magnetic stirrer for 1 h.at room temperature to obtain a homogenous mixture. Next, the CEA mixture was stirred again by sonication in an ultrasonic bath for 1 h to obtain a uniform dispersion of SMs in the base solvent. Two SMs and four types of solvents were used. The performance of the eight prepared CEAs was investigated for the treatment and recovery of fuel from oil sludge via an extraction process, as shown in Table 3.

**Table 3:** Types and compositions of CEA employed in this study.

CEA name	CEA composition
CEA1	MEK + DOWFAX
CEA2	Naphtha + DOWFAX
CEA3	Gasoline + DOWFAX
CEA4	Kerosene + DOWFAX
CEA5	MEK + STH
CEA6	Naphtha + STH
CEA7	Gasoline + STH
CEA8	Kerosene + STH

### Characterisation of WSO and recovered fuels

The WSO used in this study was obtained from Baiji refineries. It was stored under room temperature, which was maintained at approximately 23 °C. WSO was mixed well before each experiment. The properties and components of the waste sludge can be changed through each batch. Given the high difference amongst various WSO samples, the experimental data can be considered just in relative terms; various oily sludge samples will generate various data, but the same relationships must be maintained. The composition of WSO was evaluated via heating it at high temperatures to drive off the targeted oily sludge composition. WSO was heated (approximately 20 gm) to evaluate the water content at 100 °C in a muffle furnace for 2 h. The lost mass of the sample was recorded as moisture loss. The organic material contents in WSO were evaluated via heating a sample (approximately 10 g) into 650 °C for 2 h. Other oily sludge components, which consist of sediments and ashes, were weighed. All tests were repeated twice. The physical characteristics of the recovered fuel with the standard ASTM test methods used for each test were conducted in the Iraqi North Oil Company (Baiji Refineries) and the College of Petroleum Processes Engineering Laboratories/Tikrit University.

### DBBE unit

**Figure 2:**
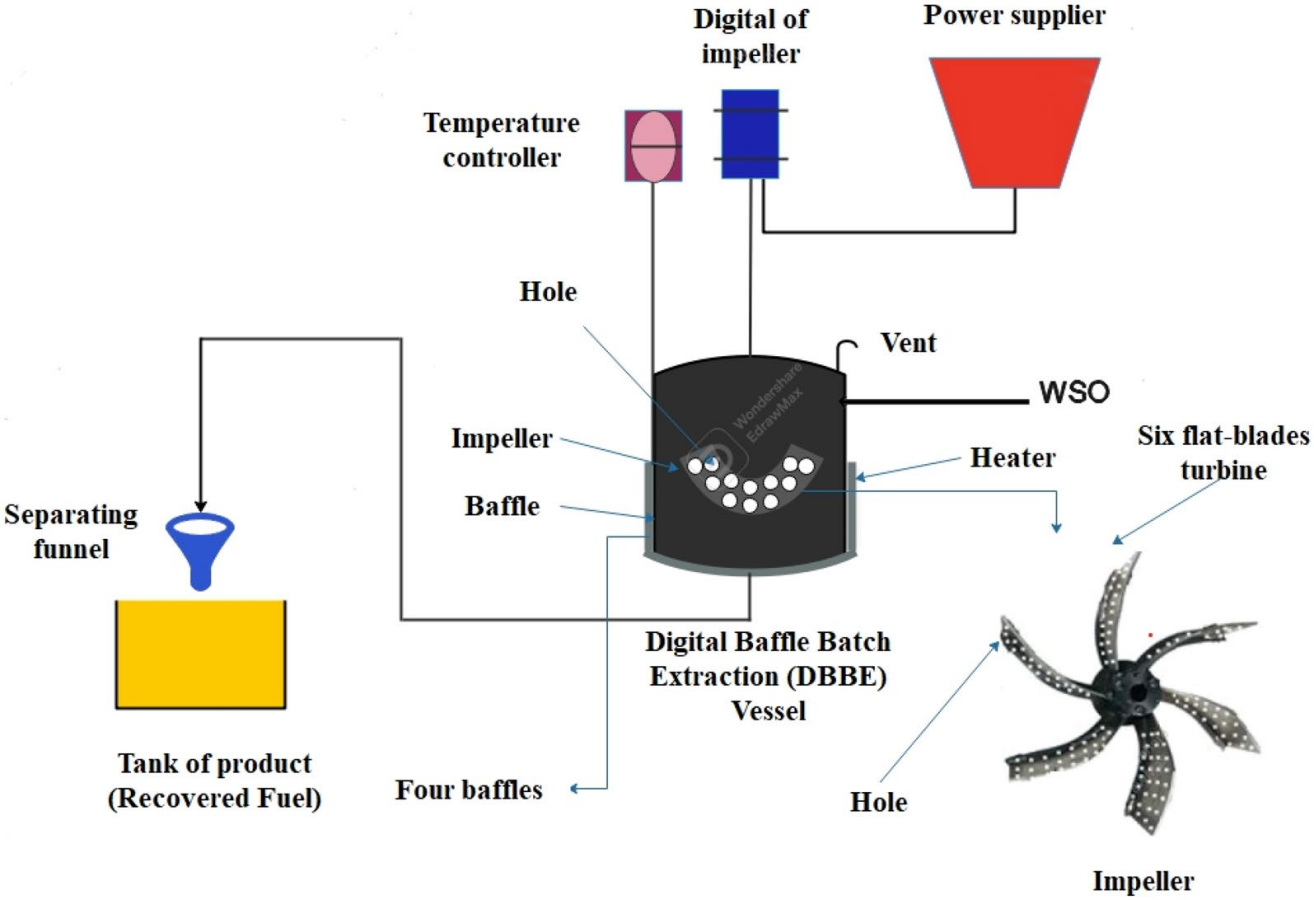
Experimental setup of the DBBE unit.

DBBE unit developed for as a new design for the oily sludge extraction process. The DBBE unit was locally designed, manufactured and installed (Fig. 2). The DBBE was installed at the Petroleum and Gas Refining Engineering Department/ College of Petroleum Processes Engineering/Tikrit University/Iraq. The geometrical dimensions of the DBBE unit are illustrated in Table 4. The used mixer in the extraction unit is small blade area type (High speed turbine mixer). Six flat blade turbines that contain circular holes that are harmonically doled out over the impeller surface in a hexagonal shape are designed to promote the mixing channels in the DBBE and improve the performance of CEA in SEP. Four baffles are installed inside the DBBE vessel in a uniform dimension as follows: 38 cm between each tow baffles, and each baffle with a 2.5 cm protrusion emerges. All unit components were manufactured from stainless steel materials. An efficient heater provided by a local market was used as a jacket that surrounded the DBBE unit. This heater can dispense the temperature from 0 to more than 500 °C. REX-C series intelligent industrial temperature controller was used to control the temperature of the unit via controlling the operation of the heater (Finglai Electric, Ltd.; China), and it can control the temperature from 0 to more than 400 °C. The DBBE was isolated using glass wool for the outer perimeter and could operate under severe experimental conditions (temperature > 1000 °C). The main part in the DBBE unit is digital mixer which was calibrated by means of a high-quality digital camera and photographing the mixer arm at different speeds, and then counting the number of rotations of the mixer arm per minute using the slow imaging feature and comparing it with its reading (RPM).

**Table 4:** The geometrical dimensions of DBBE unit.

Description	Specification (cm)
Cylinder Height, H	14.50
Cylinder Diameter, D	11.0
Diameter of impeller	9.0
Length of impeller	5.0
Depth of impeller	5.0
Width of baffle	2.50
High of baffle	20.0

### CEA process

**Figure 3:**
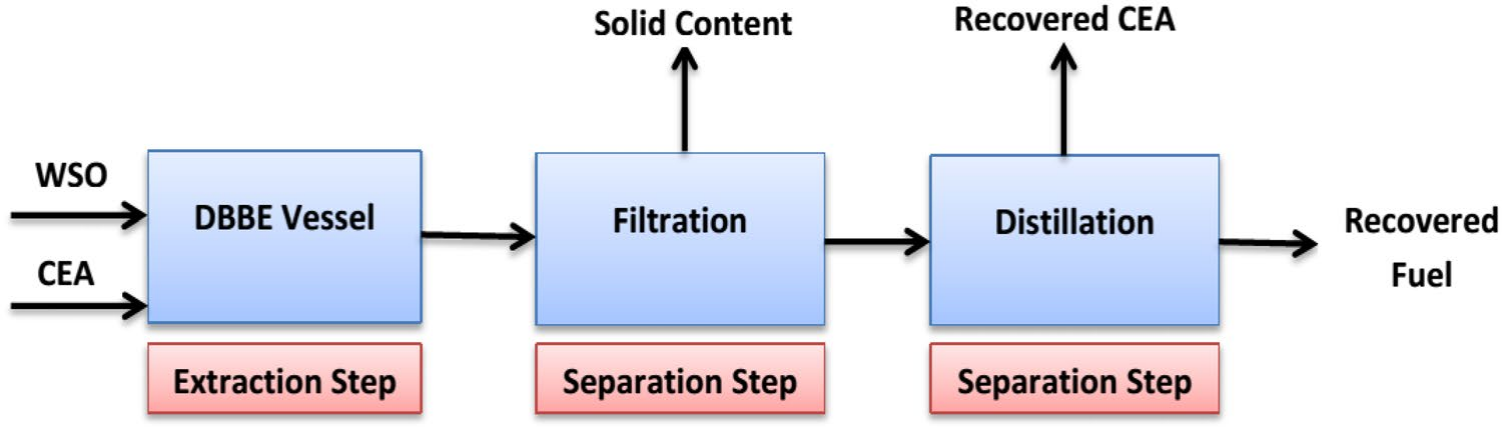
Solvent extraction process with solvent recovery.

The overall experimental steps of recovering fuel from WSO via SEP were illustrated in Fig. 3. After the preparation of CEA, it was added in 50 gm of oily sludge samples. Various CEA/sludge (1:1 – 4:1) ratios were employed. WSO – CEA mixture was mixed at various extraction times (10–20 min) and temperatures (20 – 60 °C) by employing the DBBE unit with a constant speed of 130 rpm. The applied agitation speed was selected based on the experimental setup for producing the best homogenous mixing media with minimum energy consumption. Table 5 shows the experimental variables of this study according to the full factorial experimental design: The number of experimental runs using SM was 192. Also, four runs were achieved using various solvents, without employing SM and under the optimal operating conditions to prove the significant performance of adding SM to the solvent. All experiments are conducted at atmospheric pressure. The DBBE vessel was covered well to prevent losses in CEA. After completing the experimental run under the specified conditions, the produced mixture of oil sludge and CEA was gravity-filtered using filter paper (Whatman, medium-fast). Then, the semi-solid materials were kept in a muffle furnace for drying under 80 °C for more than 24 h to remove any CEA and them weighed. The solid content in oily sludge was determined by subtracting the weight of fresh paper from the dried paper. The filtrate was fed into atmospheric distillation process using batch distillation unit. CEA was recovered as a distillate based on the distillation temperature of each type until the distillate flow decreased significantly. The applied distillation temperature of each CEA type was determined separately and experimentally using a simple distillation apparatus. The recovered CEA amount was evaluated and recorded to compare with the original amount added prior to the stirring. The distillate bottom volume (recovered fuel) was determined and recorded. The specific gravity of the recovered fuel was evaluated using the standard ASTM D 1298 method. The recovered fuel amount (by mass) was determined from the recorded volume and fuel density. The recovered fuel was determined as a mass fraction of the original oily sludge. The total amount of the produced fuel and the semi-solid materials was combined and compared with the original oily sludge mass to quantify the loss in sludge materials during the SE process. Several characteristics of the recovered fuel under the best operating conditions were evaluated by using ASTM test methods, such as the Conradson carbon residue (ASTM D 189), ash contents (ASTM D 582) and asphaltenes contents (ASTM D 3279). Also, the fuel obtained under the best conditions was tested for additional physical properties, such as flash point (ASTM D 93), water contents (ASTM D 95), viscosity (ASTM D 445), salt contents (ASTM D 3230), sulphur contents by X-ray sulphur meter (model RX-360SH, ISO 8754, ASTM D4294-03), metal contents employing inductively coupled plasma (ICP), and ASTM distillation (ASTM D 86). Next, the heavy fuel recovered under the best conditions was distilled using the distillation temperature range of the diesel fuel. Also, the physical characteristics of the produced diesel fuel were analysed using the previous standard ASTM test methods, and the diesel index (ASTM D 611) was additionally analysed. The characteristics of the produced diesel were tested to compare with the commercial diesel that was produced in Baiji Refineries.

**Table 5:** Experimental variables for SEP process in the DBBE unit.

Variable	Level	Value
CEA type	8	CEA1, CEA2, CEA3, CEA4, CEA5, CEA6, CEA7, CEA8
Extraction time, min	2	10, 20
Extraction temperature, ^0^C	3	20, 40, 60
CEA/WSO ratio	4	1, 2, 3, 4

## Results and discussion

### Characterisation of WSO

The characteristics of the oily sludge were evaluated, as illustrated in Table 6. The sediments and ashes in the WSO sample cause variations in evaluating the physical characteristics of WSO. Therefore, the standard ASTM test method was used to modify the results. WSO included water and solid contents (e.g. sediments and ashes). Also, it included low levels of volatile hydrocarbons content (low-molecular weight hydrocarbons) and high levels of non-volatile hydrocarbons, which referred to large contents of complex hydrocarbon compounds, such as content carbon residues, resins and asphaltenes. The content of hydrocarbon compounds was 70% by mass of the original oily sludge on a wet basis and 89.7% on a dry basis. This high content of hydrocarbon compounds encouraged the valuable fuel recovery from the waste sludge oil. The obtained characteristics indicated the high levels of water and solid contents. Therefore, the original sludge sample has undergone a dewatering and solid elimination step in the present work to recover valuable fuel.

**Table 6:** Characteristics of WSO.

Property	Value
Water content	21.960%
Solid content	8.040%
Volatile Hydrocarbons	10.0%
Non-volatile Hydrocarbons	60.0%
Density g/cm3 @ 15 °C	0.830
API gravity @15 °C	38.9820
Viscosity, cst @37.8 °C	297.4370

### Effect of CEA type on fuel recovery

**Figure 4:**
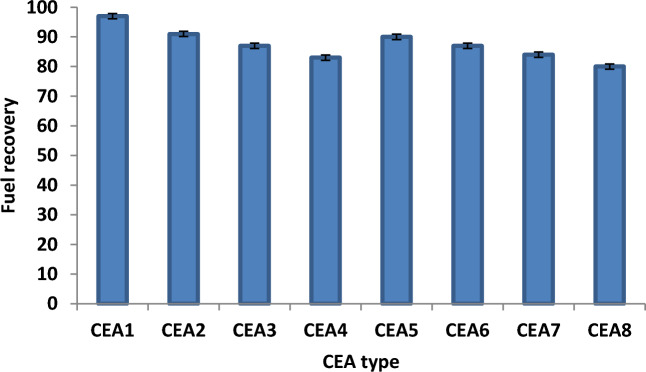
Effect of CEA type on fuel recovery under the best experimental conditions (WSO/CEA ratio of 4, time of 20 min, and temperature of 60 °C).

The effect of CEA type on fuel recovery from oily sludge under different experimental conditions was investigated. Figure 4 shows the experimental results under the optimal conditions. The experimental data proved that the fuel recovery efficiency was as follows: CEA1 > CEA2 > CEA5 > CEA3 & CEA6 > CEA7 > CEA4 > CEA8. The experimental results proved that the performance of different solvent in recovering the fuel from the real waste sludge oil was dramatically enhanced via adding the different surfactant materials. So, the results indicated that the solvent and surfactant composite improved the efficiency of fuel recovery. This behaviour can be attributed to the fact that varying the type of solvent enhanced the dissolution of crude oil based on the performance and physical properties of the solvent used^10,16,27^. Figure 4 depicts that the CEAs of MEK and Dowfax (CEA1) provide the best fuel recovery efficiency (97%) under the best conditions (WSO/CEA ratio of 4, time of 20 min, and temperature of 60 °C). The obtained results can be attributed to the fact that the solubility of crude oil was highly affected by the solvent type. The solvent used has severe polarity and decreases the viscosity of WSO for an effective and fast extraction with certain SMs^28^.

### Effect of extraction time on fuel recovery

**Figure 5:**
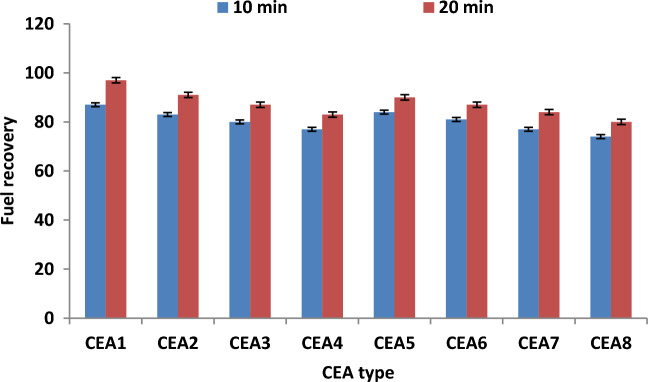
Effect of extraction time on fuel recovery under the best experimental conditions (WSO/CEA ratio of 4 and temperature of 60 °C).

The influence of extraction time on the fuel recovery 10 and 20 min was investigated. Figure 5 illustrates the experimental results of the effect of time using different types of CEAs under the best operating conditions. The results proved that the increase in extraction time improved the efficiency of fuel recovery in the DBBE unit. The fuel recovery efficiency under the best conditions (WSO/CEA ratio of 4 and temperature of 60 °C) was improved from 87 to 97%, 83% to 91%, 80% to 87%, 77% to 83%, 84% to 90%, 81% to 87%, 77% to 84%, and 74% to 80% using CEA1, CEA2, CEA3, CEA4, CEA5, CEA6, CEA7, and CEA8, respectively. The improvement in the efficiency of fuel recovery with rising extraction time can be attributed to the increase in the extraction time , which improved the number of collisions, interactions and contact time between fuel drops and CEA components and enhanced the mass transfer rate between the mixture components^25,29–33^. The higher efficiency fuel recovery was 97% at an extraction time of 20 min using MEK and DOWFAX cocktail (CEA1) under the optimal conditions.

### Effect of extraction temperature on fuel recovery

**Figure 6:**
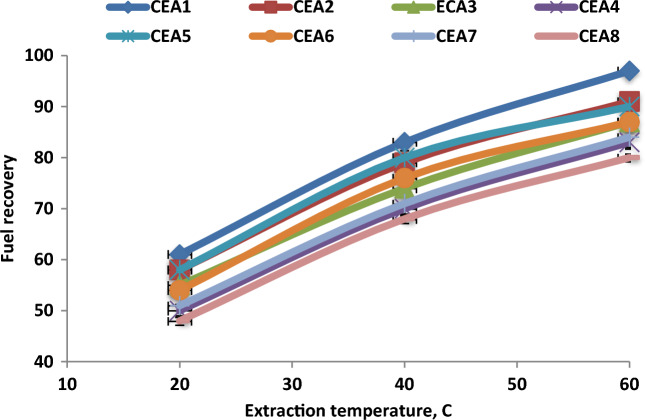
Effect of extraction temperature on fuel recovery under the best experimental conditions (WSO/CEA ratio of 4 and time of 20 min).

The experimental results of the impact of extraction temperature on the fuel recovery performance for all CEA types in the DBBE unit were presented in Fig. 6. The results showed that the efficiency of fuel recovery was improved with increasing the extraction temperatures. The increase in temperature from 20 to 40 °C enhanced the fuel recovery efficiency from 61 to 83%, and further increasing the extraction temperature to 60 °C improved fuel recoveries to 97% using CEA1 in DBBE unit. The increase in extraction temperature enhanced the space between the extraction agent molecules and reduced the viscosity of the extraction agent and oily sludge, which improved the fuel recovery efficiency. Also, an increase in temperature improved the mass transfer rate between the mixture phases and enhanced the extraction performance which significantly improved the efficiency of fuel recovery^16,30,34–36^. The results proved that the maximum fuel recovery under the extraction temperature of 60 °C was 97% under the best experimental conditions.

### Effect of CEA/WSO ratio on fuel recovery

**Figure 7:**
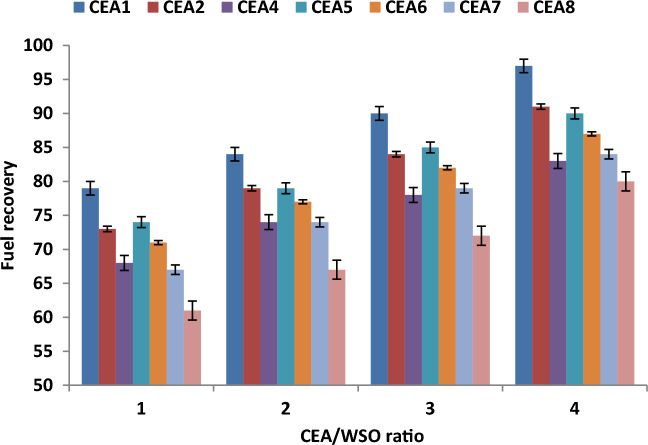
Effect of CEA/WSO ratio on fuel recovery under the best experimental conditions (extraction time of 20 min and temperature of 60 °C).

Figure 7 shows the influence of CEA/WSO ratio on valuable fuel recovery under the best experimental conditions (extraction time of 20 min and temperature of 60 °C) using different types of CEA. The experimental data proved that the increase in CEA/WSO ratio has positive effect on the efficiency of fuel recovery. So, the results showed that the recovered fuel was enhanced with the increase in the volume of used CEA. Increasing the CEA/WSO ratio from 1 to 4 improved the fuel recovery efficiency from 79 to 97% using the best CEA. This behaviour can be attributed to the increase in the volume of solvent used, which enhanced the amount of fuel that can be dissolved in it^10,16,27^. At the best ratio of 4, MEK and DOWFAX extraction agent (CEA1) provide the maximum fuel recovery (97%) under the best conditions.

### Effect of the surfactant on fuel recovery

**Figure 8:**
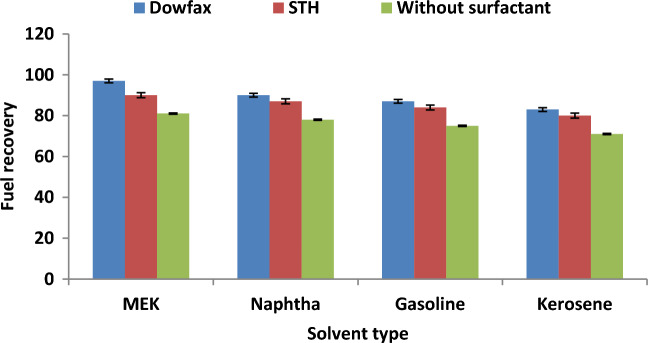
Effect of using SMs on fuel recovery under the best experimental conditions (CEA/WSO ratio of 4, extraction time of 20 min and temperature of 60 °C).

The effect of adding SMs in the extraction solvent on the fuel recovery from WSO in DBBE was studied. DOWFAX and STH were used in this study as new SMs to enhance the performance of oil treatment and recovery process. Figure 8 illustrates the performance of SEP in the presence and absence of SMs with different solvents under the optimal conditions (CEA/WSO ratio of 4, extraction time of 20 min and temperature of 60 °C). The results proved that the use of CEA via combined solvent and SM for extraction significantly enhanced the efficiency of fuel recovery from oily sludge. Figure 8 shows that fuel recovery efficiency was 81% when only MEK solvent was used, whilst the fuel recovery efficiency improved to 90% and 97% when using the CEAs of STH and DOWFAX with MEK solvent under the best experimental conditions, respectively. This behaviour can be attributed to the addition of SM, which led to the destabilisation of the interface between the discontinuous and continuous phases, thereby causing emulsion breakage, weakening the stabilisation of oily sludge and improving the extraction of fuel from oily sludge. Also, chemical surfactants are the most appropriate technique to break the crude oil emulsion and improve the droplet coalescence and agglomeration to generate larger droplets that transfer to the surface layer^37,38^. The experimental data proved that the best CEA was formed from MEK as the solvent and DOWFAX as the SM, which achieved maximum fuel recovery efficiency (97%) from WSO under the optimal operating conditions.

Finally, the results proved that the increase in extraction temperature, extraction time, CEA/WSO ratio and adding the surfactant materials into the solvent for producing the new composite of solvent and surfactant were improved the efficiency of fuel recovery from the real waste sludge oil. So that, the maximum fuel recovery rate of 97% was achieved using the best CEA (e.g. MEK and Dowfax) at a period of 20 min, temperature of 60 °C and 4:1 CEA/sludge ratio.

### Effect of CEA type on water separation

**Figure 9:**
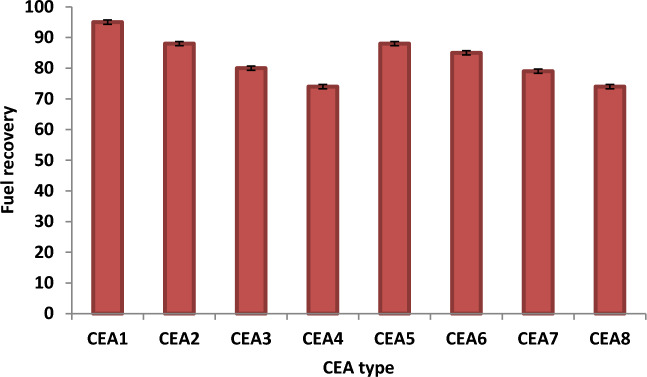
Effect of CEA type on water separation under the best experimental conditions (WSO/CEA ratio of 4, time of 20 min, and temperature of 60 °C).

Figure 9 explains the influence of the CEA on the water recovery from oil sludge. The results proved that the water separation efficiency was in the following order: CEA1 > CEA2 & CEA5 > CEA6 > CEA3 > CEA7 > CEA4 & CEA8. The results indicated that the addition of the solvent and surfactant composite enhances water separation. The water separation efficiency was the best (95%) via employing the CEAs of MEK and Dowfax (CEA1) under the optimal conditions (WSO/CEA ratio of 4, time of 20 min, and temperature of 60 °C). In general, due to the solubility enhancement of oily sludge, the improvement of water separation based on the type of solvent used mainly depends on the solvent, its performance and its important properties for extraction process. The solvent and SMs used have high polarity and decrease the viscosity of WSO for an effective and fast extraction process^28,39^.

### Effect of extraction time on water separation

**Figure 10:**
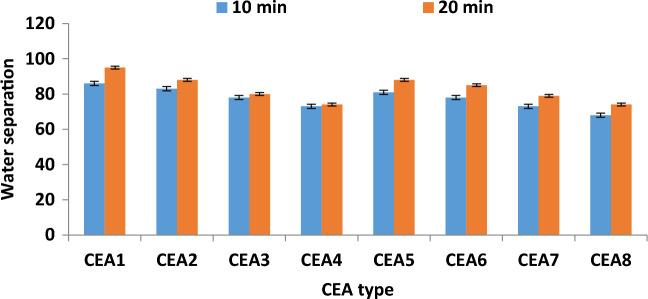
Effect of extraction time on water separation under the best experimental conditions (WSO/CEA ratio of 4 and temperature of 60 °C).

The impact of extraction time on the performance of water separation from oily sludge in DBBE unit at 10 and 20 min was studied. Figure 10 explains the results of time influence using various types of CEA under the optimal operating conditions. The increase in extraction time enhanced the water separation performance using various types of CEA under all experimental conditions. The water separation performance was enhanced from 86 to 95% using CEA1 under the best conditions (WSO/CEA ratio of 4 and temperature of 60 °C). The enhancement in the water separation efficiency with rising extraction time can be attributed to the fact that the increment of the extraction time improved the number of collisions between fuel drops and CEA components, which improves the mass transfer rate amongst the mixture components in turn. Also, enhancing the extraction time improved the contact time amongst mixture phases, thereby improving the extraction activity^25,29,30,40^. High efficiency fuel recovery of 95% was achieved by employing MEK and DOWFAX cocktail (CEA1) for 20 min under the best experimental parameters.

### Effect of extraction temperature on water separation

**Figure 11:**
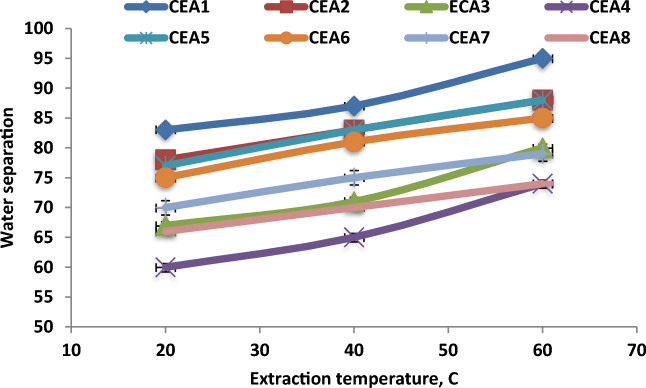
Effect of extraction temperature on water separation under the best experimental conditions (WSO/CEA ratio of 4 and time of 20 min).

The effect of extraction temperature on the water separation performance under different experimental conditions was investigated. Figure 11 shows the effect of temperature on water separation using different types of CEA under the best conditions in the DBBE unit. The experimental data explained that the water separation performance was enhanced with the increase in the extraction temperatures. Increasing the temperature from 20 to 40 °C improved the water separation performance from 83 to 87%, further enhancing the temperature to 60 °C improved water separation to 95% using CEA1 under the best conditions (WSO/CEA ratio of 4 and time of 20 min). The increase in temperature improved the space between extraction agent molecules, decreased the viscosity and density of the extraction agent and oily sludge components, and enhanced the mass transfer rate between mixture phases, which improved the water separation rate. Also, increasing the temperature improved the breaking of emulsion components and enhanced the extraction performance^16,30,34,41,42^. The increment in temperature increased the kinetic energy of water droplets and its collision led to more chances of emulsion breaking and agglomeration^43^. The results proved that the best water separation rate was 95% under the extraction temperature of 60 °C under the best experimental conditions in the DBBE unit.

### Effect of CEA/WSO ratio on water separation

**Figure 12:**
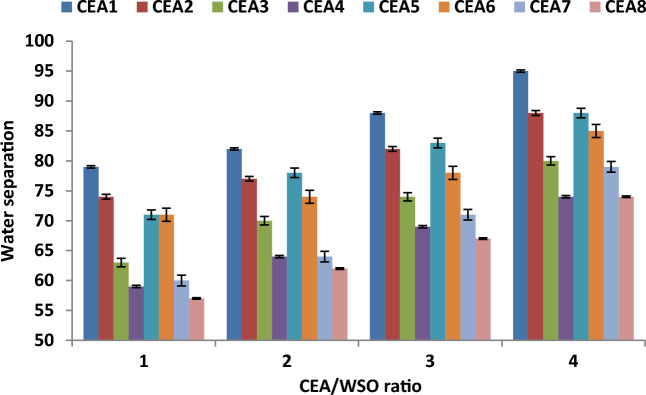
Effect of CEA/WSO ratio on water separation under the best experimental conditions (extraction time of 20 min and temperature of 60 °C).

Figure 12 explains the influence of CEA/WSO ratio on the water separation performance under the best experimental conditions (extraction time of 20 min and temperature of 60 °C) using different types of CEAs. The results proved that the water separation performance was improved with increase in the volume of CEA used. The increase of the CEA/WSO ratio from 1 to 4 improved the fuel recovery efficiency from 79 to 95% using the best CEA. This behaviour can be attributed to the increased volume of the solvent used, which enhanced the water separation^10,16,27,44^. Also, increasing the volume of the CEA used reduces the viscosity of oily sludge, which leads to the easy destabilisation of the emulsion and the enhancement of the water separation performance^9,39,45,46^. MEK and DOWFAX extraction agent (CEA1) at the best ratio of 4 provide the maximum fuel recovery (95%) under the best conditions.

### Effect of the surfactants on water separation

**Figure 13:**
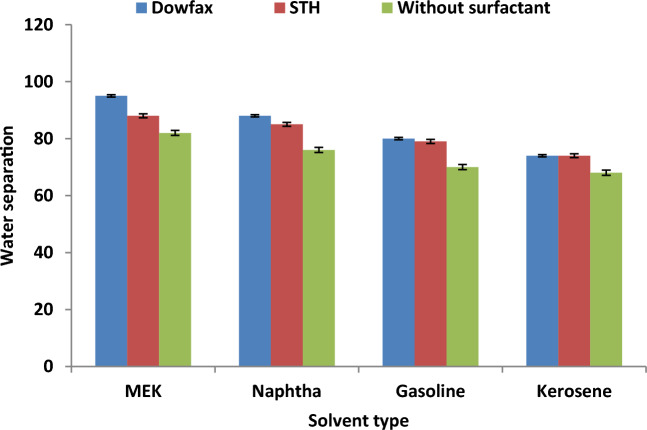
Effect of using SMs on water separation under the best experimental conditions (CEA/WSO ratio of 4, extraction time of 20 min and temperature of 60 °C).

Figure 13 explains the impact of adding SMs into the extraction solvent on the performance of water separation from WSO in the presence and absence of SMs with various solvents under the best conditions (CEA/WSO ratio of 4, extraction time of 20 min and temperature of 60 °C) in DBBE. In this study, DOWFAX and STH were selected as new SMs to enhance the performance of the extraction process. The results showed that the use of a CEA via a combined solvent and SM for the extraction process extremely improved the water separation performance from oily sludge. The results showed that the rate of water separation when only MEK solvent was used was 82%, whereas the fuel recovery efficiency improved to 88% and 95% via employing the CEAs of STH and DOWFAX with MEK solvent under the optimal conditions, respectively. This behaviour can be attributed to that addition of SM that led to the destabilisation of the interface between the discontinuous and continuous phases, thereby causing emulsion break, weakening the stabilisation of oily sludge and enhancing the separation of water droplets from oily sludge via the extraction process. In addition, the use of chemical surfactants is the most appropriate technique to break crude oil emulsion and improve droplet coalescence and agglomeration to generate larger droplets that transfer to the surface layer and enhance the rate of water separation in the DBBE unit^37,38,47^. The results proved that the best CEA was formed from the combination of MEK as the solvent and DOWFAX as the SM, which achieved the highest water separation rate (95%) from the oily sludge under the best experimental parameters.

The combination in the effect of different process variables showed that the increase in the values of temperature, time, CEA/WSO ratio and using the new composite of solvent and surfactant were enhanced the efficiency of water separation from the real waste sludge oil. Therefore, the best rate of water separation (95%) achieved using the best CEA (e.g. MEK and Dowfax) at a period of 20 min, temperature of 60 °C and 4:1 CEA/sludge ratio.

### Effect of baffle on the removal of DBT via extraction

The new extraction unit design enhanced the reactivity of the SEP, as explained in Fig. 1. The best fuel recovery efficiency and water separation rate from the WSO under the best conditions in the new DBBE unit design were 97% and 95%, respectively. The new extraction unit design prevented the natural rotation from sticking to the wall whenever the rotational speed of the impeller enhanced the mass transfer rate in the DBBE unit, thereby improving the performance of sludge oil treatment technology. SEP was used in a boost DBBE unit to determine the activity and performance of the new CEAs under different experimental conditions^48,49^.

### Assay of the recovered fuel quality

Although the amount of recovered fuel is an important parameter in the sludge oil treatment process, its quality is a basic concern. The assay of the fuel produced under the best experimental conditions (i.e. CEA1, extraction time of 20 min, extraction temperature of 60 °C and 4:1 CEA/sludge ratio) was analysed based on the standard ASTM test methods, and the obtained data were illustrated in Table 7. The results proved that the viscosity of the recovered fuel (132 cst) was much lower than that of the original oily sludge (297.4370 cst). In comparison with the original oil, the viscosity result of the recovered oil can be due to the recovery of lighter hydrocarbon compounds than heavier hydrocarbons. The ash content of the recovered fuel was 0.072 wt%, which is less than the solid content of the original WSO at 8.040 wt%. This result indicated that lower amounts of heavier and metal-containing hydrocarbons were recovered and extracted. A similar behaviour can be observed for the properties of carbon residues and asphaltene contents. The results proved that the solid separation from the oily sludge was efficient and satisfactory. According to the results that indicate the high amount of recovered fuel and the low contents of ashes, carbon residues and asphaltenes, the fuel produced by using MEK and DOWFAX as new CEA in DBBE unit under the best conditions contained higher contents of lighter hydrocarbons than heavier hydrocarbons. The water content of the recovered oil was nil compared with the high water content of the original sludge oil (21.960 wt%). The obtained water content data indicated the excellent water separation from oily sludge. The high sulphur content of the recovered fuel (2.032 wt%) indicated that the fuel needed further treatment before its used as a finished fuel^50,51^.

**Table 7:** Physical properties of recovered fuel.

Physical property	Recovered fuel
Sp. gr. @15 °C	0.8464
API @15 °C	3**5**. 6
Carbon residue, wt%	3.01
Ash content, wt%	0.072
Asphaltene content, wt%	3.12
Water content, vol%	Nil
Sulfur content wt%	2.032
Salt content, mg/L	1**5**.9
Viscosity, cst @37.8 °C	132

### Recovery of diesel fuel components

The ASTM distillation method was conducted for the recovered fuel under the best conditions (CEA1, extraction time of 20 min, extraction temperature of 60 °C, 4:1 CEA/sludge ratio). Approximately 68 vol% of the fuel produced at the boiling range of 174–347 °C was diesel fuel. The recovered oil was assessed and analysed in accordance to standard ASTM test methods. The results were compared with the assay of commercial diesel fuel from Baji refineries (Table 8)**.** Compared with the commercial diesel fuel, the produced diesel includes a larger content of carbon residues, ashes and sulphur compounds. The flash point of the recovered diesel fuel (58 °C) was lower than the value of the commercial diesel (64 °C). Also, the diesel index of the recovered diesel fuel was higher than the desirable value, which may delay ignition and lead to diesel knock problem. The density and viscosity of the recovered diesel fuel was lower than that of commercial fuel. This result can be attributed to the remarkable content of light hydrocarbons. The recovered and commercial diesel fuels in the temperature range of ASTM distillation were approximately similar. Based on the comparison between the two fuel samples, the recovered diesel oil must be fed into further treatment processes to reduce the contents of carbon residue, ash, sulphur contaminations and other physical properties before it can be used as a finished diesel oil^52,53^.

**Table 8:** Physical properties of recovered diesel fuel in comparing with commercial diesel fuel.

Physical property	Recovered diesel fuel	Commercial diesel fuel
Sp. gravity @15 °C	0.8071	0. 8173
API at @15 °C	43. 8	41. 6
Ash content, wt%	0.034	0.029
Carbon residue, wt%	0.087	0.039
Water content, wt%	Nil	Nil
Flash point, ^0^C	58	64
Total sulfur, wt%	1.73	0.83
Viscosity @ 37.8 ^0^C	1.7	2
Diesel index	61	53
Final boiling point, IBP (°C)	174	177
5%	187	191
50%	244	250
90%	311	316
Final boiling point, FBP (°C)	347	350

## Conclusion

This study focused on treating and recovering heavy oils from WSO via a SEP. Extraction technology was enhanced using new extraction agent cocktails, which consisted of four solvents, namely, MEK, naphtha, petrol, and kerosene in the presence of two new SMs (e.g. Dowfax and STH), in a DBBE unit. The solvent only used to recover the fuel from WSO in the traditional extraction process. In the present work, the new solvent and surfactant composite and the modified design of the extraction unit significantly improved the efficiency of fuel recovery from the real waste sludge oil. The influence of the most important parameters, such as the extraction time (e.g. 10 min and 20 min), extraction temperature (e.g. 20 °C, 40 °C and 60 °C) and CEA/sludge mass ratios (e.g. 1, 2, 3 and 4), on the efficiency of the extraction process were studied. By adding the baffle, SMs and the distinctive design of the extraction vessel enhanced the extraction performance for WSO. In comparison with STH, the results showed that Dowfax was the optimal SM for oil recovery from WSO using different solvents and conditions. The combination in the influence of different process variables showed that the increase in the value of all variables (temperature, time, CEA/WSO ratio and using the new composite of solvent and surfactant) improved the efficiency of fuel recovery from the real waste sludge oil. The best extraction conditions are 20 min extraction time, 60 °C extraction temperature and 4:1 CEA/sludge ratio. The extraction of MEK, naphtha, petrol and kerosene using Dowfax as the SM under optimal conditions retrieved 97%, 90%, 87% and 83% of the sludge as recovered fuels, respectively. The results showed that the best CEA (e.g. MEK and Dowfax) provided 97% fuel recovery efficiency under the optimal conditions. The physical characteristics of the recovered fuel were evaluated. The recovered fuel was distilled to generate diesel oil. The properties of the produced fuel were measured and referred to high sulphur and carbon residue contamination, as well as high diesel index. Thus, the recovered diesel fuel requires treatment processes prior to its use as finished oil.

## Data Availability

All data generated or analyzed during this study are included in this published article.

## References

[1] Che HNH (2020). Potential of *Jatropha curcas* L. as biodiesel feedstock in Malaysia: A concise review. Processes.

[2] Humadi JI (2021). Fast, non-extractive, and ultradeep desulfurization of diesel in an oscillatory baffled reactor. Process Saf. Environ. Prot..

[3] Humadi JI (2022). Dimensionless evaluation and kinetics of rapid and ultradeep desulfurization of diesel fuel in an oscillatory baffled reactor. RSC Adv..

[4] Al-Khodor, Y. A. A., & Albayati, T. M. *Real heavy crude oil desulfurization onto nanoporous activated carbon implementing batch adsorption process: Equilibrium, kinetics, and thermodynamic studies.* Chemistry Africa 1–10 (2022).

[5] Kadhum, A. T. & Albayati, T. M. Desulfurization techniques process and future challenges for commercial of crude oil products. In *AIP Conference Proceedings* (AIP Publishing LLC, 2022).

[6] Kariminezhad E, Elektorowicz M (2018). Comparison of constant, pulsed, incremental and decremental direct current applications on solid-liquid phase separation in oil sediments. J. Hazard. Mater..

[7] Hui K (2020). Status and prospect of oil recovery from oily sludge: A review. Arab. J. Chem..

[8] Zhao Y (2022). Systematical analysis of sludge treatment and disposal technologies for carbon footprint reduction. J. Environ. Sci..

[9] Motevali N (2020). Investigating Centrifuging Conditions for Sustainable Recovery of Fuel from Oily Sludge.

[10] Zubaidy EA, Abouelnasr DM (2010). Fuel recovery from waste oily sludge using solvent extraction. Process Saf. Environ. Prot..

[11] Shahbaz M (2023). A review of waste management approaches to maximise sustainable value of waste from the oil and gas industry and potential for the State of Qatar. Fuel.

[12] Chrysalidis A, Kyzas GZ (2020). Applied cleaning methods of oil residues from industrial tanks. Processes.

[13] Reynolds G (2004). Operational pollution from shipping: Sources, environmental impact and global contribution. Shipping and ports in the Twenty-first Century.

[14] Nezhdbahadori F (2018). A comparative study on the efficiency of polar and non-polar solvents in oil sludge recovery using solvent extraction. Environ. Monit. Assess..

[15] Hu G (2016). Development of Novel Oil Recovery Methods for Petroleum Refinery Oily Sludge Treatment.

[16] Al-Doury MMI (2019). Treatment of oily sludge using solvent extraction. Pet. Sci. Technol..

[17] Hu G (2020). Comparative life-cycle assessment of traditional and emerging oily sludge treatment approaches. J. Clean. Prod..

[18] Tunçal T, Uslu O (2014). A review of dehydration of various industrial sludges. Drying Technol..

[19] Kam, E. K. Assessment of sludges and tank bottoms treatment processes. In *The 8th International Petroleum Environmental Conference. November* (2001).

[20] Meng, Y. H., *et al*. Research on oily sludge treatment by solvent extraction. In *Advanced Materials Research*. (Trans Tech Publ, 2013).

[21] Mohammed MY, Albayati TM, Ali AM (2022). Imidazolium-based ionic liquids for extraction of sulfur compounds from real heavy crude oil. Chem. Africa.

[22] Mohammed, M. Y., Albayati, T. M., & Ali, A. M. The role of extractive and oxidative desulphurization techniques of fuel oils using ionic liquids: An overview. In *AIP Conference Proceedings*. (AIP Publishing LLC, 2022).

[23] Shie J-L (2004). Pyrolysis of oil sludge with additives of catalytic solid wastes. J. Anal. Appl. Pyrol..

[24] Kamla Y (2017). Effect of the inclination of baffles on the power consumption and fluid flows in a vessel stirred by a Rushton turbine. Chin. J. Mech. Eng..

[25] Jafar SA, Nawaf AT, Humadi JI (2021). Improving the extraction of sulfur-containing compounds from fuel using surfactant material in a digital baffle reactor. Mater. Today Proc..

[26] Taiwo EA, Otolorin JA (2016). Solvent blend performance in hydrocarbon recovery from Nigerian tank bottom sludge. Int. J. Eng. Technol..

[27] Khalil FI, Algawi RJ (2021). Oily Sludge Treatment by Froth Flotation: Crude Oil Recovery. Des. Eng..

[28] Negin C, Ali S, Xie Q (2017). Most common surfactants employed in chemical enhanced oil recovery. Petroleum.

[29] Li X (2012). Operational parameters, evaluation methods, and fundamental mechanisms: Aspects of nonaqueous extraction of bitumen from oil sands. Energy Fuels.

[30] Hu G (2016). Oil recovery from petroleum sludge through ultrasonic assisted solvent extraction. J. Environ. Sci. Health Part A.

[31] Khadim AT, Albayati TM, Saady NMC (2022). Removal of sulfur compounds from real diesel fuel employing the encapsulated mesoporous material adsorbent Co/MCM-41 in a fixed-bed column. Microporous Mesoporous Mater..

[32] Mohammed MY, Ali AM, Albayati TM (2022). Choline chloride-based deep eutectic solvents for ultrasonic-assisted oxidative desulfurization of actual heavy crude oil. Chem. Eng. Res. Des..

[33] Lin F (2016). Role of ethyl cellulose in bitumen extraction from oil sands ores using an aqueous–nonaqueous hybrid process. Energy Fuels.

[34] Meyer D (2006). Oil Tank Sludge Removal Method.

[35] Khadim AT, Albayati TM, Saady NMC (2022). Desulfurization of actual diesel fuel onto modified mesoporous material Co/MCM-41. Environ. Nanotechnol. Monitor. Manag..

[36] Hamad KI (2022). Enhancement of activity and lifetime of nano-iron oxide catalyst for environmentally friendly catalytic phenol oxidation process. Cleaner Eng. Technol..

[37] Al-Sabagh AM (2011). Synthesis and evaluation of some new demulsifiers based on bisphenols for treating water-in-crude oil emulsions. Egypt. J. Pet..

[38] Kralj JG, Schmidt MA, Jensen KF (2005). Surfactant-enhanced liquid–liquid extraction in microfluidic channels with inline electric-field enhanced coalescence. Lab Chip.

[39] Al-Doury MMI (2019). Treatment of oily sludge produced from Baiji oil refineries using surfactants. Pet. Sci. Technol..

[40] Humadi JI (2022). Evaluation the performance of the tin (IV) oxide (SnO_2_) in the removal of sulfur compounds via oxidative-extractive desulfurization process for production an eco-friendly fuel. Int. J. Chem. React. Eng..

[41] Fathi, M. I., *et al*. Improvement of design synthetic nano-catalysts for performance enhancement of oxidative desulfurization using batch reactor. In *AIP Conference Proceedings* (AIP Publishing LLC, 2022).

[42] Ahmed GS, Humadi JI, Aabid AA (2021). Mathematical model, simulation and scale up of batch reactor used in oxidative desulfurization of kerosene. Iraqi J. Chem. Pet. Eng..

[43] El-Naggar A (2011). Solvent extraction-gas chromatography for oil recovery from petroleum sludge using petroleum cuts. J. Am. Sci..

[44] Hu G, Li J, Hou H (2015). A combination of solvent extraction and freeze thaw for oil recovery from petroleum refinery wastewater treatment pond sludge. J. Hazard. Mater..

[45] Hu G, Li J, Zeng G (2013). Recent development in the treatment of oily sludge from petroleum industry: A review. J. Hazard. Mater..

[46] Kokal S (2005). Crude-oil emulsions: A state-of-the-art review. SPE Prod. Facil..

[47] Eow JS (2001). Electrostatic enhancement of coalescence of water droplets in oil: A review of the current understanding. Chem. Eng. J..

[48] Abbas AN, Hasan SM, Talib MA, Noori M, Cata S (2022). Formic acid dehydrogenation using noble-metal nanoheterogeneous catalysts: Towards sustainable hydrogen-based energy. Catalysts.

[49] Talib MA, Aidan MD (2014). Purification of aniline and nitrossubistituted aniline contaminants from aqueous solution using beta zeolite. Chem. Bulgarian J. Sci. Educ..

[50] Kadhum ST, Alkindi GY, Albayati TM (2022). Remediation of phenolic wastewater implementing nano zerovalent iron as a granular third electrode in an electrochemical reactor. Int. J. Environ. Sci. Technol..

[51] Talib MA, Aidan MD (2013). Shape-selective adsorption of substituted aniline pollutants from wastewater. Adsorp. Sci. Technol..

[52] Albayati TM, Doyle AM (2014). SBA-15 supported bimetallic catalysts for enhancement isomers production during n-heptane decomposition. Int. J. Chem. Reactor Eng..

[53] Talib MA, Sophie EW, Arthur AG, Aidan MD (2014). Heterogeneous alkane reactions over nanoporous catalysts. Transp. Porous Media.

